# Recent Advances in Bioengineered Scaffolds for Cutaneous Wound Healing

**DOI:** 10.3389/fbioe.2022.841583

**Published:** 2022-03-01

**Authors:** Jianghui Qin, Fang Chen, Pingli Wu, Guoming Sun

**Affiliations:** ^1^ College of Chemistry and Environmental Science, Institute of Life Science and Green Development, Hebei University, Baoding, China; ^2^ Affiliated Hospital of Hebei University, College of Clinical Medicine, Institute of Life Science and Green Development, Hebei University, Baoding, China

**Keywords:** biomaterials, wound healing, skin, bioengineered scaffolds, pro-regenerative

## Abstract

Wound healing is an evolved dynamic biological process. Though many research and clinical approaches have been explored to restore damaged or diseased skin, the current treatment for deep cutaneous injuries is far from being perfect, and the ideal regenerative therapy remains a significant challenge. Of all treatments, bioengineered scaffolds play a key role and represent great progress in wound repair and skin regeneration. In this review, we focus on the latest advancement in biomaterial scaffolds for wound healing. We discuss the emerging philosophy of designing biomaterial scaffolds, followed by precursor development. We pay particular attention to the therapeutic interventions of bioengineered scaffolds for cutaneous wound healing, and their dual effects while conjugating with bioactive molecules, stem cells, and even immunomodulation. As we review the advancement and the challenges of the current strategies, we also discuss the prospects of scaffold development for wound healing.

Bioengineered scaffolds play a key role and represent a great progress in wound healing and skin regeneration. Cutaneous wound healing is currently treated either with typical bioabsorbable scaffolds or pro-regenerative ones. Though both scaffolds facilitate wound healing, the pro-regenerative scaffolds bring about complete skin structures, while traditional biological scaffolds often lead to scarred skins. This review focuses on recent development of tissue-engineered scaffolds, especially the therapeutic interventions of pro-regenerative scaffolds for cutaneous wound healing.

**GRAPHICAL ABSTRACT:**
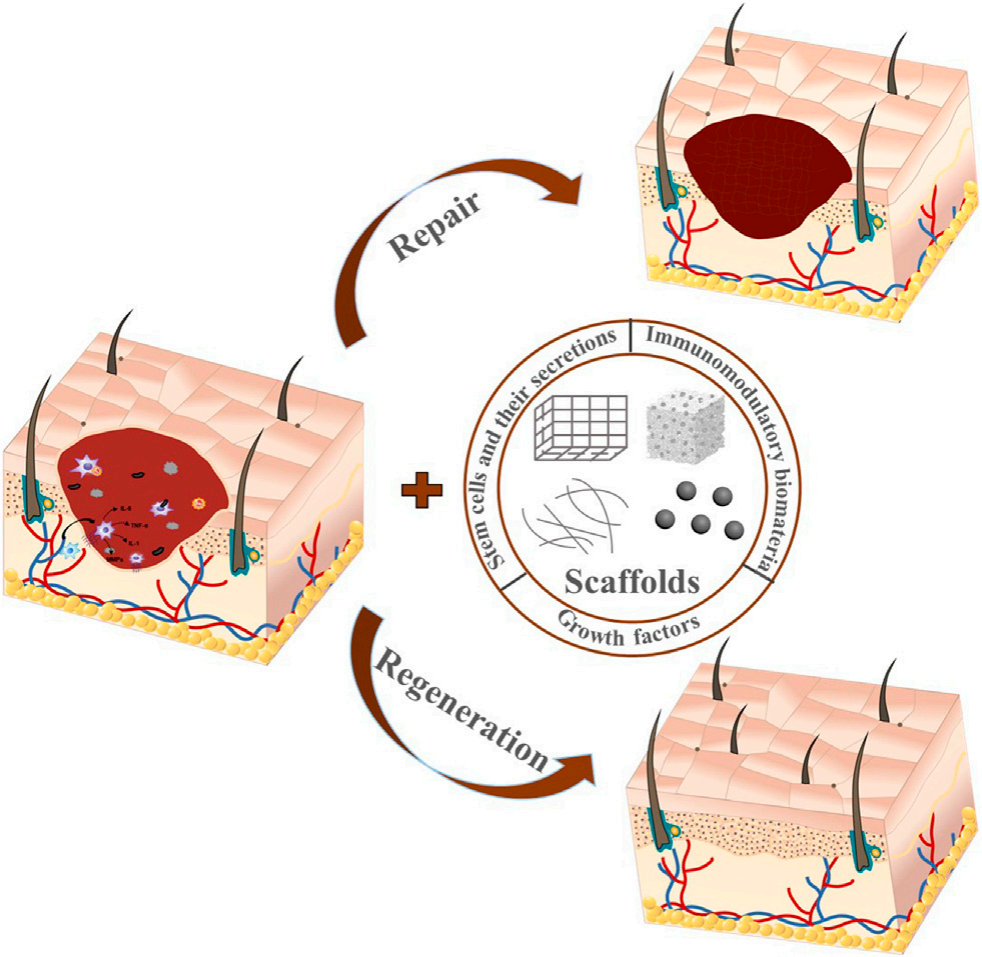


## 1 Introduction

Skin encompasses complex multilayer structures and is arguably the largest organ in the human body. Along with its appendages, the skin plays vital roles in maintaining body functions and protecting the internal organs from hostile exterior environments (Chambers and Vukmanovic-Stejic, 2020). As the outermost layer, skin is also the most vulnerable organ and could get injured easily. Superficial wounds can heal perfectly after treatment, but deep injuries can barely heal completely without proper treatments. Insufficient or untimely treatments of wounds are unable to restore normal skin and commonly lead to severe health problems, and even death. According to World Health Organization (WHO), 180, 000 deaths are caused by burns and more than 11 million cutaneous wounds require effective and timely medical attention every year (Monavarian et al., 2019). In the US, approximately six million patients struggle with chronic wounds (Norouzi et al., 2015). Appropriate and timely treatments are thus very critical. Though enormous strides have been made to decipher scarless wound healing that enabled us to develop advanced products to treat cutaneous injuries, perfect skin healing therapies remain a significant challenge (Sun et al., 2018).

Complete skin wound healing that restores full-thickness skin with its appendages is vital in rebuilding skin functions (Berthiaume et al., 2011). Skin grafting is a classical approach to treat deep skin injuries (Kim et al., 2019). The skin grafts encompass autograft, allograft, and skin substitutes. Autograft is considered the gold standard in the field of wound healing because of its non- or low-immune responses. The limited availability and the new wounds incurred all inhibit large-scale autograft transplantation (Kuppan et al., 2011; Augustine et al., 2014). Allograft skin could be an alternative choice when the autologous skin grafts are inapplicable or unavailable, but it may trigger infection and immune rejection (Parenteau et al., 2000). Xenografts may be used while neither autograft nor allograft is available (Kumbar et al., 2008; Van Lieshout, 2012), but they are mostly used as temporal protective layers before further treatments. Skin substitutes are tissue-engineered artificial skin equivalents, which were developed as alternatives. They could outperform the skin grafts, and become increasingly promising therapeutics for cutaneous wounds (Macneil, 2007; Chocarro-Wrona et al., 2019; Langer, 2019). Skin substitutes showed great potential in promoting complete wound healing (Sun et al., 2011b; Sun, 2017).

Tissue-engineered skin substitutes are primarily designed to mimic three-dimensional (3D) porous natural extracellular matrix (ECM) to create a microenvironment to enable cell proliferation and migration, thereby promoting wound healing (Hussey et al., 2018; Pina et al., 2019). The structure of biomaterial and its bioactive components determine biological properties, which are essential for the function of the scaffolds. Biomaterials that have good biocompatibility can reduce or eliminate foreign-body response, and are inclined to promote complete wound healing. Meanwhile, antibacterial scaffolds that can prevent bacterial infection and colonization are also crucial for perfect cutaneous wound healing (Hasan et al., 2017; Hasan et al., 2018). Both synthetic polymers and natural materials have been widely explored to fabricate skin scaffolds (Pina et al., 2019). In addition to biocompatibility, controllable biodegradation is also important for the property of scaffolds. The porous structure not only enables cell migration and exchanges of nutrition and wastes, but guides the phenotypic transformation of certain cells (Kim et al., 2019). With the advancement in tissue engineering and skin biology of scarless wound healing, recent attempts have been shifted from creating skin equivalents to regenerating full skin by unlocking the inherent regenerative abilities with pro-regenerative scaffolds (Gaharwar et al., 2020). Pro-regenerative scaffolds that activate the regenerative immune response show great potential in promoting full skin regeneration (Wu et al., 2021a).

Biological scaffolds lead the way in regenerative therapeutics. Immunomodulating (Wu et al., 2021a) and polysaccharide-based (Wu et al., 2021b) scaffolds were previously discussed, respectively, which is not the focus of this review. In this review, we will focus on the latest advancement in all biomaterial scaffolds for wound healing. We start with a brief introduction to the wound healing phases. We then discuss the emerging philosophy of designing biomaterial scaffolds, followed by the progress in precursor development. We pay particular attention to the therapeutic interventions of bioengineered scaffolds for cutaneous wound healing, and their dual effects while conjugating with growth factors, stem cells, and even immunomodulation. Though great strides have been made, ideal treatment remains an unmet need. We then discuss the challenges regarding the current strategies and the future perspective of scaffold development for wound healing. The ability to completely regenerate skin addresses an urgent unmet need in wound care. Engineering pro-regenerative scaffolds to restore complete skin structures will lead to clinical translational therapy in perfect skin regeneration.

## 2 Wound Healing Scaffolds

### 2.1 Wound Healing

**FIGURE 1:**
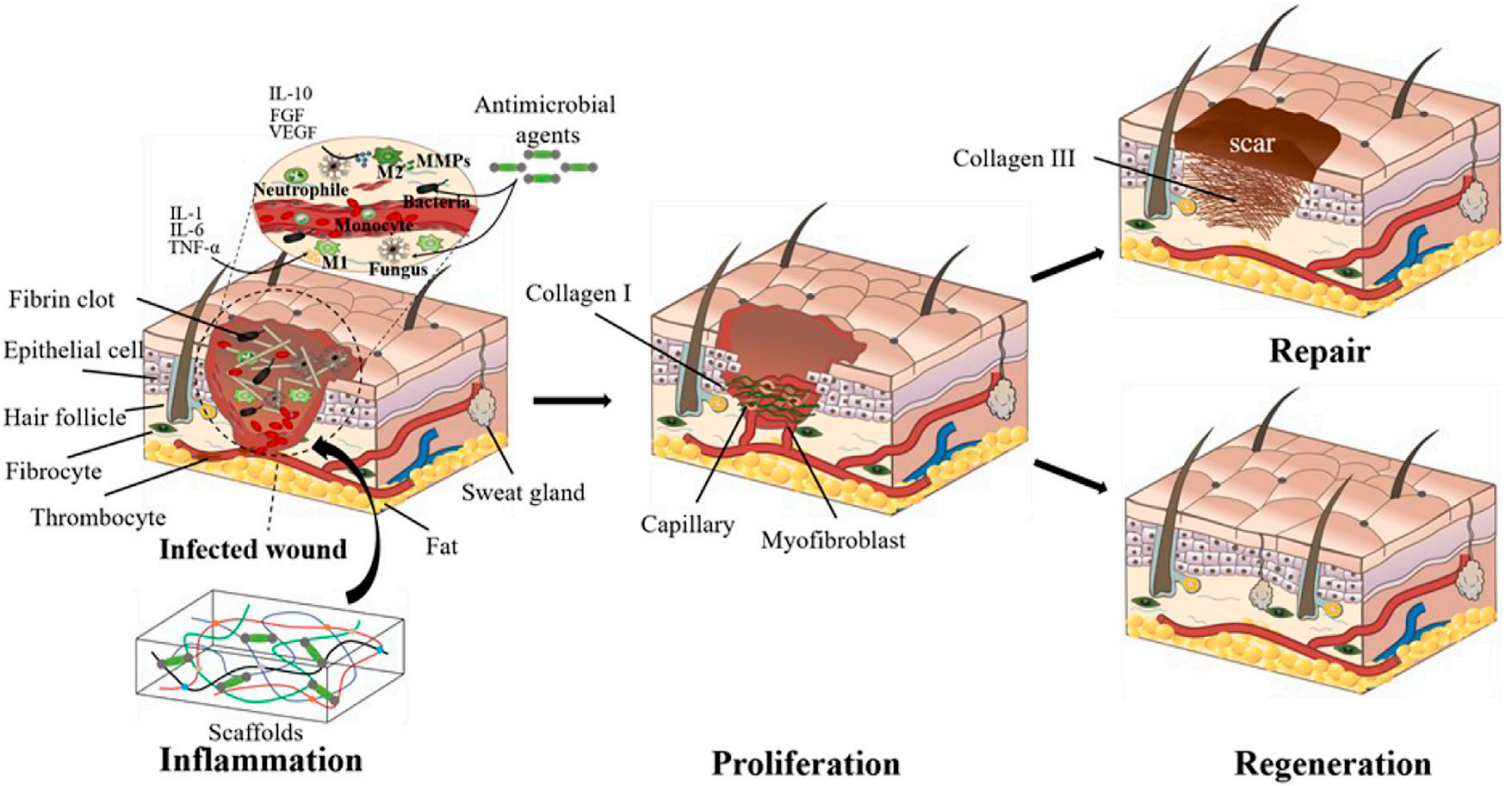
Cutaneous wound healing treated with tissue-engineered scaffolds. The wounds are currently treated either with typical biological scaffolds or pro-regenerative ones. Though both scaffolds facilitate wound healing, the pro-regenerative scaffolds bring about more complete skin structures, while the traditional biological scaffolds mostly lead to scarred skin.

Classical wound healing undergoes three distinct but continuous phases: inflammation, proliferation, and remodeling (Eming et al., 2017). Inflammation stage begins with hemostasis and the formation of platelet embolism, then the fibrin matrix consolidates into scaffolds to facilitate cell homing (Etulain, 2018). Inflammatory cells such as neutrophils and macrophages play a key role in cleaning the dead cells, bacteria, and contagious organisms (Kim et al., 2008; Gaharwar et al., 2020). However, the overexpression of inflammation response often leads to scar tissue formation (Sun et al., 2018). Bacterial infection even causes prolonged inflammation and delays wound healing, which may even become chronic wounds (Caldwell, 2020), thus leading to detrimental outcomes. Therefore, inflammation response is crucial for wound healing outcomes. The proliferation and migration of angiogenic cells to the scaffolds enable the formation of vascularized new tissues. Meanwhile, macrophages also induce and accelerate angiogenesis in the proliferation phase (Gurtner et al., 2008). The degeneration, transformation, and regeneration of ECM take place simultaneously and last months during the tissue remodeling stage (Kim et al., 2019). Cutaneous wounds can be treated with either repairing therapy or regenerative therapy. Repairing is the typical wound healing process that is usually accompanied with scar formation (Gurtner et al., 2008), while the regenerative therapy could restore the full dermal layers with skin appendages (Figure 1) (Sun, 2017; Xie et al., 2020). The recent advances in wound healing treatments give patients hope more than ever to restore scarless skin.

### 2.2 Scarless Wound Healing

In adults, the abnormal wound healing can be classified as either underhealing (e.g., diabetic wounds) or overhealing (e.g., hypertrophic scar) (Gurtner et al., 2008). Though much is still unknown about the mechanism of scarless wound healing, great strides have been made in scarless wound healing. Phan et al. reported that lymphoid enhancer-binding factor 1 (Lef1) enabled folliculogenesis in fetal mice even after a week of birth, but it is turned off after skin formation and remains quiescent in adults (Haydont et al., 2019; Phan et al., 2020). Interestingly, they also found that the successful expression of Lef1 in fibroblasts can enable adults to obtain similar skin regeneration abilities as infants. Recently, Mascharak et al. reported exciting results that preventing the activation of Engrailed-1 could inhibit scar formation and promote complete skin regeneration (Mascharak et al., 2021), while blocking mechanotransduction signaling may empower mammals with scarless wound healing.

Scarless wound healing is an evolved dynamic process and differs in species and organs, thus making it one of the most complex biological processes. Recent research has discovered many biological aspects that regulate scares wound healing. The immune cells such as macrophages, T cells, and dermal dendritic cells, which are involved in the inflammation stage, have impacts on the regeneration of skin and its appendages (Steinman, 2001; Rani and Schwacha, 2017). Our previous research showed that macrophage-modulated scaffolds could promote skin regeneration (Sun et al., 2018). Normal angiogenesis is critical to wound healing in that it ensures the transport of oxygen and nutrients to the wound site (Veith et al., 2019). Impaired angiogenesis leads to diabetic foot ulcers wounds, while robust angiogenesis results in fibrotic formation. Therefore, a novel strategy to achieve appropriate angiogenesis is very desirable for successful deep wound healing. In addition, adipocytes are also essential for scarless skin regeneration, They not only contribute to the skin stem niche, but facilitate fibroblast migration, thus promoting the regeneration of skin and its appendage (Festa et al., 2011). Shook et al. recently revealed that dermal adipocytes could regulate macrophage infiltration through lipolysis in the wound healing process, and at the same time dermal adipocytes also could be transformed into myofibroblasts to populate wounds (Shook et al., 2020). Skin appendages can be considered as a sign of skin regeneration, but also play pivotal roles in wound healing (Yildirimer et al., 2012). Hair follicles in healing wounds are necessary for the formation of normal collagen fibers, innervation of blood vessels, and adipocyte differentiation, all of which are critical for the normal function and appearance of the skin. Moreover, hair follicles contain an important reservoir of keratinocyte stem cells with established roles in maintaining skin and hair homeostasis and responding to skin insult (Sun, 2017; Monavarian et al., 2019). Though many efforts have been made to decipher the mechanism of scarless wound healing, it has yet been fully elucidated.

## 3 The Philosophy of Wound Healing Scaffolds

The progress in biomaterial science and bioengineering advanced the development of tissue-engineered scaffolds, allowed us to create a microenvironment to regulate cells and biomolecules for tissue repair and regeneration (Hollister, 2005; Sultana, 2018). The advancement of wound healing scaffolds depends greatly on the approaches of chemical or biological synthesis, modification, characterization, as well as fabrication technologies (Abdulghani and Mitchell, 2019; Gaharwar et al., 2020).

### 3.1 Polymeric Biomaterials for Scaffolds

Biomaterials are the backbone of scaffolds, and they play an essential role in the functions of the scaffolds. Currently, the scaffolds are mainly built based on natural materials, synthetic materials, and natural-synthetic hybrid materials (Liao et al., 2020; Rijal and Narmoneva, 2020). In this section, we discuss the development of polymeric biomaterials that are recently reported for scaffolds.

#### 3.1.1 Natural Polymer Materials

Natural polymer materials are widely used in the biomedical and pharmaceutical fields because of their excellent biocompatibility, degradability, and cell-cell recognition capabilities (Li et al., 2012). Naturally, existing biomaterials (e.g., polysaccharides, proteins) are the most investigated materials as bioengineered scaffolds (Negut et al., 2020). Polysaccharides, including dextran (Sun et al., 2018), chitosan (Ahmed and Ikram, 2016), and hyaluronic acid (Graça et al., 2020) are extensively explored for cutaneous wound healing because of their distinctive properties (e.g., antimicrobial activity). Polysaccharide-based biomaterials draw particular attention and are extensively fabricated into different scaffolds for Tissue Engineering and Regenerative Medicine (TERM). Recently, many studies explore the naturally existing materials for cutaneous wound healing (Table 1). For example, Gao et al. reported a biodegradable hydrogel scaffold fabricated from dextran and ε-Poly-L-Lysine (Gao et al., 2020), and they demonstrated that the hydrogel had antibacterial activities against pathogenic microbes and facilitated hemostasis in a rat liver injury model. Other than improving bioactivities, Wang et al. developed a shape adaptive dextran-based hydrogel for irregular wound healing (Wang Y. et al., 2020). The hydrogel gelated quickly to fill the wound sites regardless of wound dimension and thereby accelerated wound healing.

**TABLE 1:** Natural biomaterials of bioengineered scaffolds.

Materials	Method	Highlight	Biomedical application	Ref
Alginate	Pressurized gas expanded liquid (PGX) technology	Increased surface areas	The scaffolds can be leveraged to load clinically-relevant and highly bioavailable dosages of hydrophobic drugs in hydrogels	Johnson et al. (2020)
		High drug loadings		
		Accelerated burn wound healing		
Alginate	Microfluidic technology	Biocompatibility	The scaffolds ideally meet the requirements for different stages in a full-thickness skin wound model of rats	Shi et al. (2019)
		Biodegradability		
		Stimulate angiogenesis		
		Higher granulation tissue thickness		
Alginate/Chitosan	Interpolymer complexation	Highly porous	The scaffold helps in quick recovery from diabetic wounds by coordinating angiogenesis and inflammation	Mndlovu et al. (2019)
		Good thermal stability		
		Enhanced water uptake		
		Controlled degradation		
Chitosan/PI	Freezing/thawing approach	Low cytotoxicity	The hydrogel-based patches allowed the acceleration of wound healing in rats’ models and the complete healing	Hamdi et al. (2020)
		Drugs-control release		
		Antioxidant abilities		
		Enhanced angiogenesis		
Chitosan/OD	Chemical crosslinking	Coagulate heparinized	The scaffold has potential for hemorrhagic and infected wound healing in an infected wound model of rat skin	Du et al. (2019)
		Hemostatic activity		
		Antibacterial activity		
		Low cytotoxicity		
Chitosan/CMs	Electro-spinning	High wettability	The scaffolds provided an easy and rapid continuous large-scale industrial design strategy for natural bioresource-based wound dressing materials	Xia et al. (2020)
		Hydrophilicity		
		Gas permeability		
		Antibacterial activity		
Collagen	Chemical crosslinking	Facilitate angiogenesis	The scaffold helps in quick recovery from diabetic wounds by managing angiogenesis and inflammation	Long et al. (2020)
		Reduce inflammation		
		ECM accumulation		
		Re-epithelialization		
HA/CCS/HLC	Chemical crosslinking	Non-toxic	The scaffold had an excellent repair effect on deep second-degree burns	Lei et al. (2020)
		Biodegradability		
		Help cell proliferation		
Fibrin	Chemical crosslinking	Biocompatibility	The SVF-based full-thickness skin grafts are safe and accelerate the wound healing process	Nilforoushzadeh et al. (2020)
		Increase skin thickness		
		Promote cell migration		
Cellulose acetate	Electro-spinning	Good cell adhesion	The scaffold provides good cell adhesion and proliferation towards NIH 3T3 fibroblast and HaCaT cells	Ramanathan et al. (2020)
		Fluorescence properties		
		Help cell proliferation		
		Excellent porosity		
Cellulose/curcumin	Electro-spinning	Biocompatibility, Hydrophilicity	The scaffold confirms the potential of using sugarcane by-products in the design of scaffolds for skin tissue engineering	Ramphul et al. (2020)
		Pre-vascularization		
		Support cell growth		
CMC	*In-situ* free radical polymerization	Good thermal sensitivity	The scaffold provides a new strategy for future flexible and wearable temperature sensing devices	Pang et al. (2020)
		Excellent mechanical		
		Self-healing properties		
SF	3D bioprinting	Biocompatibility	The silk fibroin hydrogels could be used for nerve tissue engineering and wound healing	Kim et al. (2021)
		Mechanical stability		
		Printability		
SF/TA	Mixing	Adjustable viscoelasticity	The hydrogel could adhere to the skin surface as a flexible wearable strain sensor	Zheng et al. (2021a)
		Antibacterial properties		
		Self-healing		
Sf/borosilicate	UV crosslinking	Enhanced angiogenesis	The hydrogel could be used to regenerate diabetic wounds	Pang et al. (2021)
		Antibacterial properties		
		Reduce inflammation		

PI, protein isolate; OD, oxidized dextran; CMs, cellulose membranes; HA, hyaluronic acid; CCS, carboxylated chitosan; HLC, human-like collagen; SVF, stromal vascular fraction; CMC, carboxymethyl cellulose; TA, tannic acid; SF, silk fibroin.

Natural polymers have many active functional groups, which allow us to chemically or biologically modify them to gain even more desirable functions to achieve enhanced wound healing (Negut et al., 2020). Liang et al. recently reported a dual-dynamic-bond crosslinked hydrogel based on the quaternized chitosan for wound healing (Liang et al., 2021). The quaternized chitosan hydrogel that has good water solubility and antibacterial activity inhibited wound infection and effectively promoted wound closure. Interestingly, Zhang et al. developed an intelligent microneedle scaffold from polyvinyl acetate (PVA) and gelatin methacryloyl (GelMA) for wound healing (Zhang X. et al., 2020). The microneedle scaffold, loaded with black phosphorus quantum dots and hemoglobin, showed excellent photothermal conversion ability that gave rise to near-infrared light-responsive oxygen release to enhance chronic wound healing. Turner et al. reported core/shell vascularized 3D constructs for wound care (Turner et al., 2020). The scaffold was composed of gelatin methacryloyl, succinylated chitosan, and dextran aldehyde. The core/shell scaffold improved the viability of human bone-marrow-derived mesenchymal stems cells and human umbilical vein endothelial cells and promoted wound closure rate. Though modifying natural polymers offer many opportunities, over-modification may compromise its innate biological properties, and an appropriate modification should be taken into consideration.

#### 3.1.2 Synthetic Materials

Though natural biomaterials have great properties, they cannot meet all requirements for wound healing. Compared with natural biomaterials, synthetic biomaterials that can be tuned with desirable biological and physical properties for enhanced wound healing are extensively studied (Dong et al., 2009; Dinarvand et al., 2012). Polyester-based synthetic materials, such as polycaprolactone (PCL) and poly (lactic acid-co-glycolic acid) (PLGA), have been approved by FDA and found many applications in skin tissue engineering (Table 2). Li et al. developed a nanocomposite hydrogel that exhibited excellent injectability and self-healing behavior (Li et al., 2020). The injectable self-healing hydrogel showed robust antibacterial activity, and significantly enhanced diabetic wound healing and skin regeneration by promoting angiogenesis and neovascularization. Additionally, Xi et al. reported a hybrid nanofibrous scaffold with excellent anti-inflammatory, antibacterial, and antioxidative activities (Xi et al., 2020). They demonstrated that the multifunctional hybrid scaffold inhibited bacterial infection and accelerated chronic wound healing by restoring blood vessels. Recently, synthetic ceramic biomaterials (e.g., calcium phosphates and bioactive glasses) are also extensively investigated for wound healing. Ceramic biomaterials have excellent biodegradability, bioactivity, and electrical activity (Saxena et al., 2021a; Saxena et al., 2021b; Das et al., 2021), which makes them great candidates for TERM. Though originally used to repair hard tissues (Saxena and Pandey, 2021), they have now found applications in skin tissue engineering (Mazzoni et al., 2021). Ceramic biomaterials can regulate cell proliferation and spreading, and mediate the secretion of growth factors that promote wound healing and skin regeneration (Yu et al., 2016). Niu et al. reported a bioactive Si–Ca–P–Mo glass-ceramic nanoparticle for improved wound healing (Niu et al., 2021), for instance, and they demonstrated that the nanoparticle along with molybdate nanocrystals prominently reduced inflammation, but effectively promoted vascularization. Unlike polymeric biomaterials, though ceramic biomaterials have many potentials (Punj et al., 2021; Solanki et al., 2021), they have yet been fully investigated for wound healing.

**TABLE 2:** Synthetic materials of bioengineered scaffolds.

Materials	Method	Highlight	Biomedical application	Ref
PVA/HNT	Dispersion mixing technique	Biocompatible	The PVA-based nanocomposite wafers can provide new and suitable wound dressings for wounds exposed to infection such as burn wounds	Mohebali et al. (2020)
		Anti-bacterial		
		Anti-inflammatory		
PVA/DPHC	Freezing thawing method	Anti-bacterial	The PVA/DPHC hydrogels have great potential for use in wound dressings	Kim et al. (2020)
		Strong wound healing effect		
PCL/SPC	Electro-spinning technology	Vascularization	The scaffold could potentially be used as an envisioned approach for the efficient recovery of chronic diabetic wounds	Zehra et al. (2020)
		Compact ECM		
		Up-regulation of HIF-1α		
PCL/Alaptide/L-Arginine	Electro-spinning	Re-epithelization	The modified nanofibrous membranes are promising for treating wounds with large damaged areas	Mikeš et al. (2020)
		Improved wound closure		
P (TA)/p (HEMA)	Cryo-gelation technique	Antibacterial	The scaffold can be used as wound dressing material since it possesses antioxidant, antimicrobial, and blood compatibility properties	Sahiner et al. (2017)
		Biodegradability		
		High hemostatic		
AgNPs/pSBAA	Physical crosslinking	Germicidal	The novel non-sticky and antimicrobial zwitterionic	Huang et al. (2017)
		Higher water content	The scaffold has the potential for the treatment of infected chronic wounds	
		Low cytotoxicity		
PVP/Cipro	Electro-spinning	Antibacterial	The scaffold showed promising wound resorption characteristics in a full-thickness excisional skin wound healing mice model	Contardi et al. (2017)
		Plasticity		
		Wound resorption		
PVP/PVB	Electro-spinning	Antibacterial	The scaffold produced by the *in situ* electrospinning have the potential as a wound dressing	Liu et al. (2018)
		Air permeability		
PNIPAM/PAA	Chemical crosslinking	Stiffness tunable	Regulating scaffold’s stiffness affect therapeutic effects in the wound healing	Chen et al. (2016)
		ECM remodeling		

HNT, halloysite nanotubes; SPC, sodium percarbonate; DPHC, diphlorethohydroxycarmalol; PAA, poly (amidoamine); p (HEMA), poly (2-hydroxy ethyl methacrylate); AgNPs, silver nanoparticles; pSBAA, poly (sulfobetaine acrylamide); Cipro, ciprofloxacin; PVB, poly (vinyl butyral); PVP, polyvinylpyrrolidone; PNIPAM, poly (n-isopropyl acrylamide).

Synthetic materials alone, however, are often accompanied with inflammatory responses (Luttikhuizen and Harmsen, 2006) and relatively low bioactivity (O’brien, 2011), which are still unable to promote perfect cutaneous wound healing, and they often couple with additional components to improve the properties.

#### 3.1.3 Natural-Synthetic Hybrid Materials

As aforementioned, both natural and synthetic materials have their unique advantages and disadvantages. Synthetic materials can be manufactured precisely and consistently as designed, showing minimal variability. Synthetic materials, however, especially their degraded byproducts, may lead to proinflammatory responses and cause undesirable results (Luttikhuizen and Harmsen, 2006). Natural materials, on the contrary, are biologically compatible with many tissues or organs and are greatly explored for wound healing (Brown et al., 2012). To take advantage of the properties of both natural and synthetic polymers, hybrid materials are thereby constantly developed (Table 3). Natural-synthetic hybrid materials that consist of desirable properties of both materials could overcome the shortcomings of each material, and have found a greater application in the field of cutaneous wound healing (Sheikholeslam et al., 2018).

**TABLE 3:** Natural-synthetic hybrid materials of bioengineered scaffolds.

Materials	Method	Highlight	Biomedical application	Ref
Chitosan/PLGA	Chemical crosslinking	Low cytotoxicity	Improved wound healing when used in the diabetic rat model	Viezzer et al. (2020)
		Reduce inflammation		
		Improve neovascularization		
CMCS/SA	EDC/NHS crosslinking	Antibacterial activity	The scaffold inhibited bacteria growth and promoted wound healing in the burn-infection model	Mai et al. (2020)
		Rapid epithelialization		
		Higher collagen deposition		
Alginate/PVA	Solvent casting method	Good mechanical properties	The alginate-based hydrogel membrane could be an efficient wound healer for faster wound healing	Abbasi et al. (2020)
		Sustained release		
		Granulation tissue formation		
Dextran/HA/PEI	UV light crosslinking	Biocompatible inhibiting inflammation promoting microvascular	The hydrogel system can be considered as a promising wound dressing for the treatment of various types of wounds	Wang et al. (2019)
PVA/Dextran-aldehyde	Freeze-thaw method	Large exudate absorption	The hydrogel scaffold accelerated wound healing in full-thickness skin defect model	Zheng et al. (2019)
		Suitable transmission rate biocompatibility		
PEG/fibrin	Co-polymerize	Properties adjustable	The macroporous and mechanically reinforced fibrin-based sequential IPN hydrogels useful for dermal tissue regeneration	Gsib et al. (2020)
		Cellular infiltration		
		Tissue remodeling		

CMCS, carboxymethyl chitosan; SA, sodium alginate; HA, hyaluronic acid; PEI, polyethyleneimine; PEG, polyethylene glycol.

The emergence and development of hybrid materials allow us to tailor their compositions and tune their properties to enhance tissue repair and regeneration (Hussey et al., 2018). To take advantage of two network systems, Chen et al. developed an interpenetrating network scaffold based on agar and hydrophobically associated polyacrylamide (HPAAm) (Chen et al., 2015). As the HPAAm network could stand stress and rebuild network structure, the hybrid scaffold had remarkable self-healing property and excellent mechanical strength. Liu et al. reported a composite sponge for methicillin-resistant *staphylococcus* aureus-infected wound healing (Liu W. et al., 2021). The scaffold was prepared by mixing sodium polyacrylate (PAAS), double quaternary ammonium salts-conjugated chitosan (QAS-CS), and collagen (COL) in an aqueous solution. PAAS could promote blood coagulation by absorbing a large amount of blood and tissue fluid, QAS-CS have the inherent antibacterial effect and COL could enable cell proliferation and promote tissue reconstruction. As a result, the composite sponges showed an outstanding antibacterial and hemostatic performance, and the scaffold also enabled a robust angiogenesis and blood vessel maturation in the MRSA-infected wound. Similarly, Fathi et al. reported a hybrid electrospun scaffold from PVA, chitosan, and silk fibrous mat for wound healing (Fathi et al., 2020), in which PVA provided excellent strength and elongation properties, while chitosan and silk enhanced cell affinity through cell surface receptor ligands. They demonstrated that these three materials showed a synergistic effect on enhancing wound healing outcomes. Though combining different materials could greatly improve the scaffold properties, additional chemical modification may enable further enhancement.

Incorporating functional groups into synthetic or natural polymers is an effective way to improve the properties of the scaffolds. Xue et al. reported a gelatin-PCL (GP) nanofibrous scaffold encapsulated with black phosphorus nanosheets to improve wound treatment (Xue et al., 2021). They demonstrated that conjugating RGD (Arg-Gly-Asp) components into the scaffold promoted cell adhesion, while the GP scaffold that was loaded with doxorubicin could be heat-triggered and release doxorubicin *in situ*, thereby enhanced wound healing. Pang et al. reported an *in situ* photo-crosslinked hydrogel from borosilicate and silk fibroin, which were both chemically modified with methacryloyloxy groups in advance (Pang et al., 2021). They demonstrated that the silk fibroin -methacryloyloxy-borosilicate hydrogel could fully spread to the wound surface and firmly adhered to the wound, and protected the wound from external contamination. They further revealed that the hydrogel inhibited inflammation, but improved angiogenesis via interaction between hypoxia-inducible factor 1-alpha (HIF-1α) and Cu2^+^, thereby promoting wound healing. Liu et al. reported a thioether grafted hyaluronic acid nanofibrous hydrogel scaffold formed *in situ* for chronic wounds (Liu et al., 2020). The scaffold grafted with thioethers could effectively scavenge the reactive oxygen species (ROS) in the early inflammation phase. They proved that the scaffold was safe and effective in treating the methicillin-resistant *staphylococcus* aureus-infected wound.

Collectively, hybrid materials with a wide range of properties can thereby be achieved by changing their ratios, molecular weights, and chemical structures. Increasingly more hybrid biomaterials show great potentials and are developed into bioengineered scaffolds for cutaneous wound healing applications.

#### 3.1.4 Decellularized Materials

Decellularized scaffold materials that are capable of repairing and regenerating new tissues are also extensively studied for wound healing. Decellularization is the process of removing the cellular components that would result in immunological rejection, while preserving the morphology, 3D structures, and composition of the extracellular matrix (Cui et al., 2019). Therefore, the scaffolds made of decellularized materials have many advantages in wound healing, in terms of effectively promoting cell adhesion, migration, and proliferation (Dussoyer et al., 2020). The decellularization procedure is generally be accomplished through physical, chemical, and enzymatic methods (Bernhardt et al., 2015; Wang C.-H. et al., 2020).

The scaffolds decellularized with different methods have varied characteristics, and they can be utilized directly or refabricated into new scaffolds. Wang et al. prepared a collagen matrix for wound healing from decellularized porcine skin using supercritical carbon dioxide (SCCO_2_) technique (Wang C.-H. et al., 2020). When applied to the porcine full-thickness skin wound model, the scaffold showed good biocompatibility, low inflammation, and promoted epithelial regeneration. Gholipourmalekabadi et al. developed a hybrid wound healing scaffold fabricated from decellularized human amniotic membrane and electrospun nanofibrous silk fibroin (Gholipourmalekabadi et al., 2018). The scaffold was further seeded with adipose-tissue-derived mesenchymal stem cells (ADSCs) and evaluated in a full-thickness murine burn wound. They found that the scaffold significantly reduced fibrosis formation by releasing growth factors and recruiting inflammatory cells to the scaffold. Some antigens in the animal tissue cannot be removed by the decellularization process and may lead to immune rejection after transplantation. Therefore, the researchers used transgenic technology to edit key genes of donor animals to obtain a donor without immune rejection. Morris et al. used decellularized thrombospondin (TSP)-2 knockout mice skin to treat diabetic wounds (Morris et al., 2018), and the scaffold promoted fibroblast migration and significantly accelerated diabetic wound healing, indicating that genetically engineered materials have great pro-regenerative potentials.

Coupling decellularized materials with other components (e.g., growth factors, cells) could bring even more possibilities for full-thickness skin wound healing. Kuna et al. developed a novel composite gel by combining decellularized pig skin with human peripheral blood mononuclear cells (hPBMCs) (Kuna et al., 2017). The gel, along with angiogenic cells, promoted neovascularization, enhanced dermal collagen deposition and transformation, and further facilitated epidermal layer closure, thereby leading to improved wound healing. Adipocytes play important roles in regenerating the skin and its appendages. Chen et al. reported a hydrogel scaffold developed from the human decellularized adipose matrix (hDAM) and examined it for chronic wound healing (Chen et al., 2021). The hDAM hydrogel that was pre-encapsulated with human adipose-derived stem cells (hASCs) promoted hASC adhesion, proliferation, and migration. Furthermore, they demonstrated that the scaffold could enhance the regenerative potential of hASCs, and accelerated wound healing in a full-thickness diabetic mouse model.

### 3.2. Tissue Engineered Scaffolds for Wound Healing

The progress of wound healing scaffolds has greatly advanced the therapeutic interventions. Translational efforts to advance laboratory research into clinical practice have led to commercial products. There are many commercial products used in the clinic, but only a few, such as Integra^®^, Apligraf^®^, and Dermagraft^®^ stand out (Berthiaume et al., 2011). Integra^®^ is a semi-bilayer membrane scaffold that consists of bovine collagen and glycosaminoglycans (Halim et al., 2010). Integra^®^ could be used for deep partial- and full-thickness burns, but it often needs additional skin graft. Apligraf^®^ is composed of dermal collagen and keratinocytes, which could be used for venous and diabetic ulcers (Pham et al., 2007). Though it fastens wound healing time, Aligraf^®^ suffers short shelf life. Dermagraft^®^ is a cell sheet that grows human neonatal dermal fibroblasts on a polyglactin mesh (Halim et al., 2010). It not only requires weeks of cell culture, but is unable to repair wounds in cases where cells have limited repairing potentials (Sun, 2017). Therapeutic efficacy and physiological function of commercial wound healing products are far from meeting clinical needs, and current products are not cost-effective, which also prevent them from being widely used (Berthiaume et al., 2011; Yu et al., 2019). Therefore, it becomes very desirable to develop clinically effective wound care products to improve wound healing.

The goal of biological scaffolds is to recreate a physiological microenvironment similar to what natural ECMs do, to promote TERM (Rahmati et al., 2018). The development of scaffolds not only depends largely on the advancement of biomaterials, but they have also made great strides with the emergence of new fabrication technologies. The most used technologies in fabricating bioengineered scaffolds encompass electrospinning technology, 3D bioprinting technology, microfluidic technology and stem cell technology. In this part, we discuss primarily the recent progress in electrospun scaffolds, hydrogel scaffolds, 3D printing scaffolds for either acute or chronic wound healings.

#### 3.2.1 Electrospun Scaffolds

Electrospinning is a widely used approach to prepare nano- and micro-sized non-woven fibers through electrostatic forces driven by high-voltage electric fields (Liu et al., 2017). The electrospun scaffolds have many characteristics, such as structural similarity to the natural ECM, which could be beneficial to skin wound healing (Koosha et al., 2019; Mahanty et al., 2020). Also, the electrospinning scaffolds provide a relatively high surface-to-volume ratio and could enhance hemostasis, promote absorption of skin wound exudates (Zahedi et al., 2010). Moreover, such characteristics of electrospun fibers as the diameter, pore size, surface area, permeability, mechanical integrity, and porosity, all have a significant impact on wound healing (Liang et al., 2020). Therefore, increasingly more research has been carried out to develop micro-/nano-scale electrospinning scaffolds for deep wound healing and other medical applications (Afsharian and Rahimnejad, 2021).

The wound dressings made by electrospinning could enhance hemostasis, promote absorption of skin wound exudates and attenuate scar formation (Zahedi et al., 2010). Meanwhile, electrospun scaffolds can deliver bioactive molecules to the wound sites (Gao et al., 2019; Afsharian and Rahimnejad, 2021). Lee et al. reported a coaxial sheath-core nanofibrous loaded with platelet-derived growth factor (PDGF) and bioactive antibiotics for infectious wounds (Lee et al., 2020). The authors found that the scaffold could sustainably release PDGF for 3 weeks, which significantly promoted angiogenesis and accelerated wound healing. Interestingly, Jafari et al. developed a nanofiber scaffold with double layers, (Jafari et al., 2020), of which the top layer was loaded with amoxicillin and the bottom layer containing zinc oxide. They revealed that the scaffold had a sustained release of amoxicillin for up to 144 h, and the drug together with the scaffold accelerated wound healing and reduced scar formation. Recently, *in situ* electrospinning is also proved an effective approach for wound healing (Dias et al., 2016). Dong et al. prepared a personalized dressing with a portable electrospinning device for skin wound healing (Dong et al., 2016). The *in-situ* electrospinning dressing loaded with silver nanoparticles gives the scaffolds good antibacterial properties, and the scaffolds can achieve broad-spectrum antibacterial by slowly releasing silver ions. It is worth to note that the personalized dressing could be suitably used in medical emergencies and home disease treatment.

#### 3.2.2 Hydrogel Scaffolds

Hydrogels are 3D crosslinked hydrophilic polymer networks and are broadly used for both acute and chronic cutaneous wound healing (Asadi et al., 2021). Hydrogels are structurally similar to natural ECM, and have many exceptional capabilities, such as super water retention, excellent biocompatibility, and the ability to absorb excessive exudate, which are ideal scaffolds for wound healing (Koehler et al., 2018; Xiang et al., 2020).

Hydrogels are prepared either via chemical covalent crosslinking (e.g., radical reaction) or physical crosslinking (e.g., hydrogen bonding) (Sharma and Tiwari, 2020). The physically crosslinked hydrogels usually have self-healing and shear-thinning properties, but relatively low structural stability (Muir and Burdick, 2021). Li et al. reported a physically crosslinked antibacterial hydrogel scaffold that promoted full-thickness skin wound healing (Li et al., 2021). The hydrogel was prepared from acrylic acid, 1-vinyl-3-butylimidazolium, COOH-modified gum arabic, and aluminum chloride, in which the 1-vinyl-3-butylimidazolium promoted the self-healing of hydrogels by accelerating the migration of aluminum ions. Unlike physically crosslinked hydrogels, chemically crosslinked ones usually necessitate catalysts or initiators to produce covalent bonds, and the hydrogels have higher structural stability in both *in vitro* and *in vivo* (Muir and Burdick, 2021). Liu et al. reported a hydrogel based on Schiff-base linkage for diabetic wounds (Liu P. et al., 2021). The HA-based hydrogel is injectable, self-healing, and tissue-adhesive, exhibited excellent stability, and provided long-term protection for diabetic wounds, and further promoted angiogenesis by releasing M2 macrophage-derived exosome sustainably.

Hydrogels fabricated from many materials via different methods, have very distinct properties that suit them for different types of wounds. Those hydrogels with good mechanical properties and tissue adhesion could be used for joint skin wounds, for instance, while the self-healing hydrogels loaded with anti-inflammatory biomolecules would be more efficient in treating chronic wounds, and the hydrogel with shear-thinning and removable properties may be suitable for burn wound treatment (Zhang A. et al., 2020). Peng et al. developed a hydrogel dressing with fast gelation and good water absorption capacities for hemostasis wound healing (Peng et al., 2021). The dressing could gelate *in situ* within 4 s, and showed excellent wet adhesive properties. A hemostasis experiment further showed that the dressing could stop bleeding in approximately 10 s in a rat liver bleeding model, making it a promising scaffold for acute bleeding wound care. Interestingly, Tu et al. reported a dynamically crosslinked graphene oxide hydrogel from peptides and polydopamine (Tu et al., 2021). They took advantage of regenerative immunotherapy, and demonstrated that the hydrogel could upregulate M2 macrophage, which enabled anti-inflammation and stimulated angiogenesis, thereby significantly improving diabetic wound healing. Recently, Zheng et al. reported a smart hydrogel fabricated from polyacrylamide-quaternary ammonium chitosan-carbon quantum dots-phenol red system to monitor and treat wound healing (Zheng K. et al., 2021). The hydrogel had antibacterial activities, and its coupling with pH-responsive carbon quantum dots and phenol red indicator enabled it to detect the pH changes of the wound, thereby indirectly monitoring the real-time wound healing process through the change of color in a noninvasive way.

#### 3.2.3 3D Printing Scaffolds

3D bioprinting is a rapidly developing technology, which empowers us to prepare biological scaffolds that mimic the native tissue microenvironment for TERM (Bracaglia et al., 2017). 3D bioprinting, in which cells and growth factors are preloaded in the gels and printed into biological scaffolds, has been increasingly investigated for skin tissue engineering (Matai et al., 2020). Siebert et al. demonstrated that a 3D bioprinting scaffold loaded with VEGF enhanced anti-inflammatory response, angiogenesis, and cell proliferation at the wound site, and achieved the fastest wound healing compared with blank control (Siebert et al., 2021). A 3D bioprinting scaffold that can regulate cells accurately by precisely controlling the structure of the scaffold undoubtedly enhances wound healing. Cheng et al. reported an *in-situ* formation of biomaterial scaffold with handheld instrument to treat full-thickness burns (Cheng et al., 2020). Enhanced re-epithelialization and dermal cell repopulation were observed in the wound bed after MSC-containing fibrin bioink was directly printed onto burn wounds, thereby facilitating full-thickness wound healing. Hakimi et al. reported a handheld *in-situ* skin printer that enabled the formation of skin tissue sheets with different structures and compositions (Hakimi et al., 2018). The mixture of alginate, fibrin, collagen, and hyaluronic acid was pre-loaded with dermal and epidermal cells, and their deposition onto inclined and irregular wound surfaces achieved enhanced wound healing on both murine and porcine models, which they believe may find many applications for non-regular wounds. Maintaining cell viability within sterile scaffolds remains a challenge, and there is a long way to go to translate 3D bioprinted cell-laden scaffolds into clinics (Wu et al., 2021b).

### 3.3 Approaches to Engineer Wound Healing Scaffolds

As bioengineered scaffolds could regulate cellular functions and facilitate the exchanges of nutrients and wastes during deep wound healing, they are the most effective artificial skin substitutes of the 3D framework in recent years (Jeschke et al., 2020). The efficacy of wound healing scaffolds relies on both the material properties and the scaffold architectures and progressed with the more understanding of wound healing. Based on the wound healing mechanism, many different scaffolds were developed to treat cutaneous wound healing (Negut et al., 2020). In this part, we focus on the recent advancement of bioengineered scaffolds by integrating immunomodulatory biomaterials, bioactive molecules, and stem cells into the scaffolds.

#### 3.3.1 Immuno-Engineering Pro-Regenerative Scaffolds

The integration of immunomodulatory biomaterials or biomolecules could change the immune microenvironment to direct endogenous cells for tissue repair and regeneration (Gaharwar et al., 2020). Macrophages are the most important immune cells with multiple phenotypes, and they play an important role during the entire wound healing process (Sun, 2017; Sun et al., 2018), particularly in the inflammation stage. Macrophages can be polarized into two distinct phenotypes, i.e., M1 and M2, in which M1 macrophage produces pro-inflammatory cytokines and M2 releases growth factors (Barrientos et al., 2008; Delavary et al., 2011). That being the case, macrophages could lead to fibrotic tissue (M1) or regenerate new tissue (M2) (Martinez et al., 2008; Brancato and Albina, 2011). Therefore, immune-engineering biological scaffolds that can effectively modulate the macrophage polarization and differentiation may completely change the wound healing progress (Tylek et al., 2020).

**FIGURE 2:**
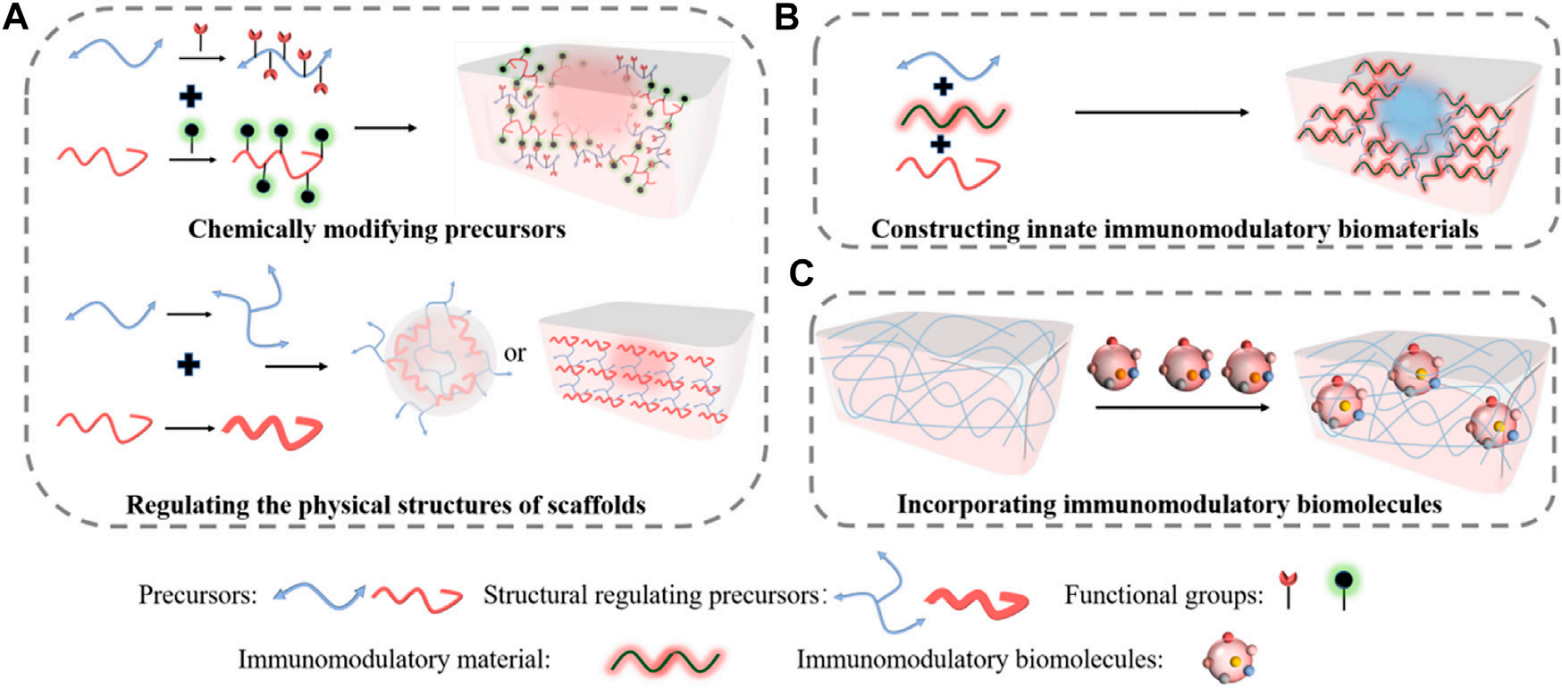
Immuno-engineering pro-regenerative scaffolds for wound healing. **(A)** Either chemically modifying precursors or regulating the physical structures enable the immunomodulatory properties of the scaffolds. **(B)** Constructing innate immunomodulatory biomaterials into pro-regenerative scaffolds for skin regeneration. **(C)** Incorporating immunomodulatory biomolecules into scaffolds to empower the regenerative capacities for wound healing.

Modifying the biochemical and structural characteristics of the macromolecules enables us to manipulate the immunomodulating property of the scaffolds (Figure 2A). Corradetti et al. reported a collagen scaffold modified by chondroitin sulfate to stimulate a pro-regenerative environment (Corradetti et al., 2017). They found that incorporating anti-inflammatory chondroitin sulfate macromolecule into the scaffold could help recruit M2 phenotype macrophage and promote neoangiogenesis. Similarly, Shen et al. synthesized a sulfated chitosan-based hydrogel that reduced proinflammatory M1 macrophages and promoted revascularization, which greatly improved diabetic wound healing (Shen et al., 2020). Our previous study also demonstrated that incorporating functional groups into dextran allowed us to tune its immune responses to upregulate M2 phenotype macrophages, thereby our dextran hydrogel regenerated full skin structures with appendages on both acute wounds and pre-existing scars (Sun, 2017). Other than modifying molecular structures, tuning the physical structures allows additional immunomodulation. Recently, Won et al. 3D printed a microchanneled PCL scaffold, and it showed great potential for wound healing (Won et al., 2020). Compared with traditional 3D printed scaffolds, the hierarchically structured scaffold could modulate macrophage polarization into M2, reduce inflammatory responses, and promote angiogenesis and stem cell homing, thereby enhancing wound healing than typical 3D printed scaffolds.

Other than immunomodulation, manipulating physical structure can generate favorable microenvironmental cues to promote skin repair and regeneration. Yin et al. developed a three-dimensional hydrogel with controlled stiffness (Yin et al., 2021), in which they demonstrated that regulating the stiffness could promote cell migration. Meanwhile, Jin et al. fabricated thin films with different topological structures prepared by electrospinning (Jin et al., 2021). They found that manipulating the topological structures of these membranes could help recruit monocytes and induce angiogenesis, thereby enhancing cutaneous wound healing. In our prior study, we also demonstrated that manipulating the pore size and biodegradation rate by changing the crosslinking density allowed angiogenic cell homing and diffused excessive inflammatory cells (Sun et al., 2010; Sun et al., 2011a; Sun et al., 2018), which greatly promoted full skin regeneration. Collectively, regulating the chemical and physical properties of the implantable biomaterial scaffold could promote regenerative wound healing.

Unlike modifying the structures of the scaffolds that require multiple steps of modifications, engineering innate immunomodulatory biomaterials could be more efficient in constructing pro-regenerative scaffolds (Figure 2B). Bioactive glass (BG) is a human-made material that has been widely studied in TERM (Gorustovich et al., 2009). Dong et al. revealed that BG could stimulate cell migration to the wound area, and it also affected macrophage polarization (Dong et al., 2017). Recently, Zhu et al. further demonstrated that an injectable hydrogel fabricated from BG and sodium alginate (SA) enhanced skin regeneration by shifting macrophage polarization from M1 phenotype into M2 phenotype. The gel was physically crosslinked through Ca^2+^ interactions with SA. The wound closure rate of normal mouse treated with BG/SA hydrogel and SA solution was faster than that of the macrophage-depleted mouse, but no significant difference was observed in the macrophage-depleted mouse treated either with BG/SA hydrogel or SA solution, indicating that shifting of M2 macrophages was essential for wound healing. The H&E staining further showed that the BG/SA hydrogel lead to more complete tissue regeneration than the SA solution in a full-thickness normal mouse wound model.

Encapsulating immunomodulatory biomolecules into intelligent responsive scaffolds is also an efficient approach to direct macrophage differentiation to improve wound healing (Figure 2C). Saleh et al. developed an adhesive hydrogel scaffold loaded with miR-223 5p mimic (miR-223*) for wound healing (Saleh et al., 2019). The scaffold was fabricated from gelatin methacryloyl and miR-223* encapsulated in hyaluronic acid-based nanoparticles. The scaffold loaded with miR-223* could accelerate wound healing by up-regulating the polarization of macrophages to the M2 phenotype. Similarly, Wu et al. reported a hydrogen sulfide (H_2_S)-releasing hydrogel for wound repair (Wu et al., 2019). They demonstrated that the hyaluronic acid (HA) hydrogel scaffold could reduce inflammation and improve wound remodeling effects in a cutaneous wound model, in which releasing H_2_S induced the expression of M2 macrophage phenotype. Meanwhile, Griffin et al. synthesized D-peptide crosslinked microporous annealed particle hydrogel (D-MAP) scaffold to activate an adaptive immune response for regenerative wound healing (Griffin et al., 2021). They also demonstrated that the immunomodulating scaffold promoted full-thickness skin regeneration in a murine model.

As more details about the interplay between immune response and wound healing are revealed, more efficient immunomodulating scaffolds will be developed, and this has become the most promising therapy to regenerate full skins. Additional discussions about the development of immunomodulating scaffolds were recently presented elsewhere (Wu et al., 2021a). Immuno-engineering has become one of the most important approaches to develop pro-regenerative scaffolds for wound healing.

#### 3.3.2 Incorporating Growth Factors Into Scaffolds

Growth factors are essential for the wound healing process in that they stimulate cell proliferation, facilitate cell migration to wound sites, and self-assemble cells into functional tissues (Greenhalgh, 1998). Incorporating bioactive molecules into the scaffolds would help create a regenerative microenvironment to promote complete wound healing. Such growth factors as vascular endothelial growth factor (VEGF) and platelet-derived growth factor (PDGF) can effectively promote vascularization, which is critical for the outcome of deep wound healing (Lai et al., 2014; Li et al., 2015). However, the short half-life, and poor release profiles of these growth factors limit their *in vivo* applications. The scaffolds serve as carriers of the controlled release and also protect the growth factors from being denatured. Xiao et al. developed a sulfobetaine methacrylate (SBMA) hydrogel and incorporated fibroblast growth factor-2 (FGF2) for full-thickness skin wound healing (Xiao et al., 2021). The *in vitro* release profile of FGF2 showed that SBMA hydrogel could increase the sustained release and maintain the bioactivity of the growth factor, and the *in vivo* data further revealed that SBMA hydrogel could promote granulation tissue formation, collagen deposition, and angiogenesis by sustaining the release of FGF2. Recently, Siebert et al. prepared a composite hydrogel loaded with VEGF for full-thickness skin wound healing (Siebert et al., 2021). The composite hydrogel was modified with light-sensitive tetrapodal zinc oxide (t-ZnO) microparticles, and VEGF release could be spatiotemporally controlled via light exposure, thus promoting angiogenesis and wound healing. Moreover, growth factors could also play synergistic roles with scaffolds to guild cell growth into functional tissues or organs (Klimek and Ginalska, 2020). Shao et al. self-assembled hyperbranched polyaminoglycoside into nanoparticles that were loaded with plasmid-encoded epidermal growth factor (EGF) and rose bengal to treat infected wound healing (Shao et al., 2021). The antibacterial rose bengal, along with EGF and the scaffold not only inhibited bacterial growth and enhanced vascularization, but also showed a synergistic effect on promoting the healing of the infected wounds. Altogether, incorporating growth factors into scaffolds significantly improves wound healing.

Typical, particularly homogenous scaffolds are usually unable to achieve spatiotemporally controlled release of several growth factors. To improve release efficiency and enhance cutaneous wound healing, multilayered scaffolds are thereby developed to deliver multiple growth factors simultaneously. As multilayered scaffolds resemble the skin structure, they can be further engineered to deliver specific growth factors at each layer based on wound healing phases (Jeckson et al., 2021). Additionally, multilayered scaffolds also have enhanced physical and biological properties, thereby they become promising scaffolds to improve cutaneous wound healing. Multilayered scaffolds are mostly fabricated through 3D printing, electrospinning, lyophilization technologies (Fu et al., 2020). The complex hierarchical scaffolds loaded with growth factors (e.g., VEGF) or therapeutic drugs (e.g., antibacterial agents) can thus promote angiogenesis and granulation (Xie et al., 2013), cell proliferation and migration, as well as collagen deposition and epithelialization (Vakilian et al., 2021). Though multilayered scaffolds have become a promising approach to treat injuries, overengineered complex scaffolds may be avoided, which could compromise their therapeutic efficacy and make the manufacture challenging.

#### 3.3.3 Encapsulating Stem Cells Into Scaffolds

Stem cells are unspecialized cells that can self-renew and differentiate into multiple cell types (Kosaric et al., 2019). Stem cells and progenitor cells can get involved in direct tissue formation or release growth factors to promote tissue repair and regeneration. When applied in cutaneous wound healing, the stem cells can reduce inflammation, promote granulation tissue formation and neovascularization (Ghieh et al., 2015). Stem cell therapies are thereby extensively investigated in wound healing.

**FIGURE 3:**
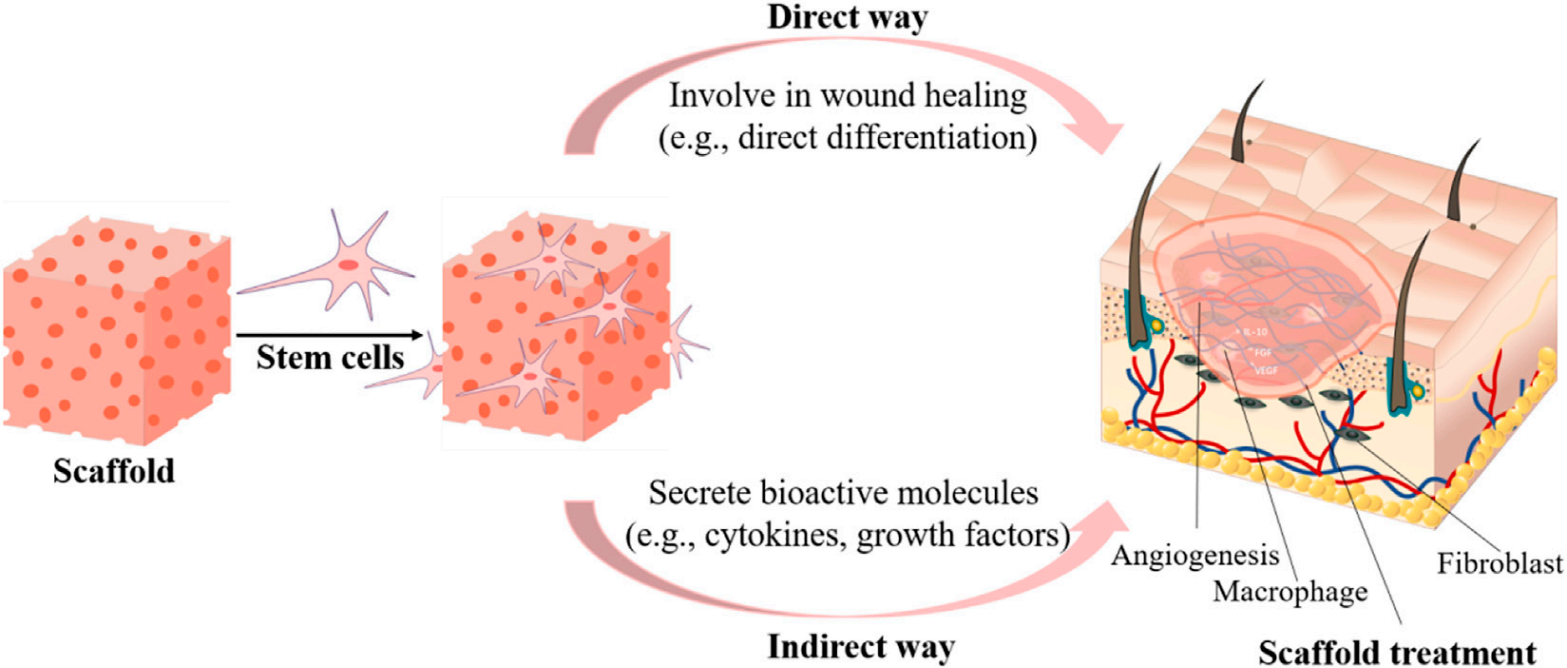
Encapsulating stem cells into scaffolds to enhance wound healing either through contributing to new tissue formation or by releasing regulating biomolecules.

Cell transplantation therapy usually has low viability *in vivo* due to poor vascularization. Encapsulating stem cells into biologically active scaffolds not only improves cell survival rate, but also plays a much more important role in TERM (Eom et al., 2015; Kwak et al., 2018). The encapsulated stem cells could promote wound healing via different procedures (Figure 3). They could form new tissues or differentiate into the desired cell types to form new tissues to promote skin regeneration. Putra et al. developed an HA-based hydrogel to support human endothelial colony-forming cells (ECFCs) for vascularization. They demonstrated that tuning the biodegradation of HA hydrogel promoted the vascular formation and enabled ECFCs integration into mouse vasculature, thereby enhancing wound healing in a burn model (Hanjaya-Putra et al., 2013). To regenerate sweat glands, Yao et al. developed an alginate/gelatin hybrid hydrogel encapsulated with mesenchymal stem cells (MSCs) and tested in a mouse burn model (Yao et al., 2020). They demonstrated that tuning the physical and biochemical cues of scaffolds could synergistically direct MSC differentiation into multiple cell lineages, which coordinately promoted sweat gland regeneration. Similarly, Roshangar et al. reported a 3D bioprinted gel scaffold from collagen and alginate with adipose-derived stem cells (ADSCs) for burn wound healing (Roshangar et al., 2021). The scaffold increased cell adhesion and proliferation. The immunohistochemistry (IHC) Staining confirmed that the scaffold facilitated ADSC differentiation into keratinocytes, and the scaffold significantly promoted wound contraction and epithelization of burn skin in rat model than cell-free treatment.

Though cells could be directly involved in tissue repair and regeneration, maintaining the viability and phenotypes remains a great challenge. Increasingly more studies indicate that stem cells also contribute to wound healing through the paracrine release of bioactive molecules (Baraniak and Mcdevitt, 2010). Zheng et al. developed a MSC-laden microgel assembled from silk nanofibers to modulate MSC paracrine actions for scarless wound healing (Zheng et al., 2020). Tuning the physical cues of this injectable gel enabled higher secretion of ANGPT-1, VEGF-α, SDF-1, and HGF, which greatly promoted vascularization, cell recruitment, tissue ingrowth, and immunomodulation, thereby leading to complete skin regeneration. Lu et al. developed a hydrogel from gelatin and silk fibroin loaded with ADSCs and platelet-rich plasma (PRP) to treat pressure ulcer wounds in mice (Lu et al., 2021). They found that the hydrogel promoted proliferation, migration, and survival time of ADSCs, which enabled the prolonged release of many angiogenic growth factors. Along with PRP that contains multiple angiogenic growth factors and facilitates fibroblast proliferation, the hydrogel scaffold achieved accelerated wound healing by reducing inflammatory infiltration, promoting angiogenesis and collagen deposition.

Though stem cells have great potentials in treating various wounds, and the cell phenotype changes could lead to undesirable outcomes during *in vivo* applications, it remains a significant challenge to be used in the clinic (Breitbach, 2007). It is worth to note that cell-free stem cell technology is increasingly investigated for TERM. Stem cells-derived small extracellular vesicles (sEVs) are nanometer membranous vesicles released by various stem cells, and they have great potentials in promoting tissue regeneration (Henriques-Antunes et al., 2019; Wang and Thomsen, 2021). Recently, Shen et al. loaded bone marrow-derived mesenchymal stem cells sEVs into a bilayered hydrogel, from which they demonstrated that the gel was capable of promoting angiogenesis and collagen deposition, and thereby accelerated wound healing (Shen et al., 2021).

Encapsulating stem cells into scaffolds certainly facilitates the reconstruction of the wound healing microenvironment, but maintaining the sterile and hydrated cell-embedded scaffolds in the wound beds is very challenging during the entire wound healing period. This would not only complicate the surgery procedure, but will also increase the cost. A more efficient and easily operated scaffold is still desirable to turn stem cells into effective wound healing therapy.

## 4 Future Perspective

Wound healing is an evolved dynamic biological process, and varies from different species. It also differs from one organ to another, and deteriorates with age. Though many studies have been carried out to improve skin tissue engineering during the last few decades, the entire mechanism of cutaneous wound healing, especially scarless skin regeneration, is yet fully understood. Incomplete understanding limits our capability to develop more efficient scaffolds to treat various wounds. Significant breakthroughs will still depend on further uncovering the myth of scarless wound healing and the advancement of scaffold fabrication technology. Though much has been revealed, wound healing is far more complicated than what has been uncovered. Each stage has its distinct characteristics for chronic and acute injuries among different ages of patients, respectively. Current theories are unable to explain all the myths behind the entire wound healing process. The advancement of wound healing treatment is still dependent on new findings of skin biology, which empowers us to design the most pro-regenerative scaffolds for complete skin regeneration.

Perfect skin regeneration is the ultimate goal for all cutaneous injuries, but only limited success achieved to date. The diversity of wound type and body location often makes the treatment even more challenging. Treatment of large skin defects or deep injuries (e.g., third-degree burns) with tissue engineered scaffolds remains a great challenge. Robust neovascularization is absolutely critical for the transportation of cells, nutrition, oxygen, and waste in repairing or regenerating large skin injuries, but it induces excessive inflammation and compromises wound healing outcomes. Clinically, transplanting and maintaining a large viable tissue engineered scaffold is nearly impossible, and large skin injuries still have to undergo multiple surgeries. As a result, an efficient scaffold with ease of handling and customization will be very desirable. Moreover, to attenuate scar formations, an ideal scaffold should also have enhanced bioactivities such as antibacterial and anti-inflammatory activities (Jeschke et al., 2020).

Integrating functionalities into the scaffolds and manipulating both chemical and physical structures allow us to fabricate more personalized wound scaffolds, especially with the advancement of fabrication methods. Dual or multiple scaffold systems, even from the same biomaterials, could achieve an enhanced synergistic wound healing effect when combined. Multifunctional scaffolds will undoubtedly play a greater role in wound healing than single functional scaffolds, but over-engineering would make manufacturing very challenging and increase the cost, which should be avoided. Meanwhile, integrating either growth factors or stem cells into scaffold improves the wound healing efficacy, but it brings about more challenges to transform scaffolds into clinical practice and obtain FDA approval.

Novel fabrication approaches help build more efficient architectures of scaffolds indisputably, but the scaffolds for perfect skin regeneration and repair may still remain a challenge until uncovering further mechanisms of wound healing. Despite all the challenges, with the collaboration among chemists, engineers, scientists and surgeons, more promising pro-regenerative scaffolds would be translated into clinical applications.

## 5 Conclusion

The therapeutic interventions of bioengineered scaffolds for cutaneous wound healing have made great strides from repairing processes to regenerative ones, but the challenge for ideal treatment still remains. Biomaterial scaffolds play a significant role in wound healing, and have been extensively investigated. Scaffolds depend on the biomaterial properties and their architectures. Understanding the philosophy of designing biomaterial scaffolds enabled us to construct scaffolds from basic crosslinking, electrospinning, 3D bioprinting to cell-matrix interactive and immunomodulating scaffolds, in which they may encapsulate cells and/or biomolecules. As such, bioengineered scaffolds could serve dual or multiple functions to promote skin wound repair and regeneration. Scaffolds built on the base of regenerative immunology show great regenerative capacity and could be the solution for many skin diseases or injuries. The advancement of wound healing rests heavily on biomaterial science and skin biology. Further exploration in skin wound healing mechanisms and novel approaches to fabricate scaffolds will lead to more clinically effective products in treating deep dermal injury and attenuating scar formation.
